# Protective effects of *Akkermansia muciniphila* on cognitive deficits and amyloid pathology in a mouse model of Alzheimer’s disease

**DOI:** 10.1038/s41387-020-0115-8

**Published:** 2020-04-22

**Authors:** Zihao Ou, Lulu Deng, Zhi Lu, Feifan Wu, Wanting Liu, Dongquan Huang, Yongzheng Peng

**Affiliations:** 1 Department of Laboratory Medicine, Zhu Jiang Hospital, Southern Medical University, 510282 Guangzhou, Guangdong China; 2 Jiangmen Central Hospital, Affiliated Jiangmen Hospital of Sun Yat-sen University, 529000 Jangmen, Guangdong China; 3 Transfusion Medicine, Zhu Jiang Hospital, Southern Medical University, 510282 Guangzhou, Guangdong China

**Keywords:** Bacteria, Lipid-storage diseases

## Abstract

**Objective:**

Alzheimer’s disease (AD) is a global health problem without effective methods to alleviate the disease progression. Amyloid β-protein (Aβ) is widely accepted as a key biomarker for AD. Metabolic syndromes, including obesity and insulin resistance, are key high risk factors for AD. *Akkermansia muciniphila* (Akk), the only representative human gut microbe in the genus *Verrucomicrobia*, can prevent the weight gain caused by a high-fat diet, repair the damaged integrity of the intestinal epithelium barrier, reduce endotoxin levels in blood and improve insulin resistance. The aim of this study is to explore the impact of Akk administration in AD model mice in different diets.

**Methods:**

APP/PS1 mice were fed either a normal chow diet or a high-fat diet and were treated with Akk by gavage each day for 6 months. The impacts of Akk on glucose metabolism, intestinal barrier and lipid metabolism in the mouse model of AD were determined. Changes in brain pathology and neuroethology were also analyzed.

**Results:**

Akk effectively reduced the fasting blood glucose and serum diamine oxidase levels, and alleviated the reduction of colonic mucus cells in APP/PS1 mice. After treatment with Akk, the APP/PS1 mice showed obviously reduced blood lipid levels, improved hepatic steatosis and scapular brown fat whitening. Moreover, Akk promoted the reduction of Aβ 40–42 levels in the cerebral cortex of APP/PS1 mice, shortened the study time and improved the completion rate in Y-maze tests.

**Conclusion:**

Akk effectively improved glucose tolerance, intestine barrier dysfunction and dyslipidemia in AD model mice. Our study results suggested that Akk could delay the pathological changes in the brain and relieve impairment of spatial learning and memory in AD model mice, which provides a new strategy for prevention and treatment of AD.

## Abstract (web summary)

APP/PS1 mice display alterations in physiological processes after treated with Akk. It is showed that the damaged integrity of the intestinal epithelium barrier is repaired, leading to obviously reduced blood glucose and lipid levels, improved hepatic steatosis, insulin resistance and scapular brown fat whitening, which are beneficial to reduce Aβ 40–42 levels in the brain of APP/PS1 mice.

## Graphical abstract

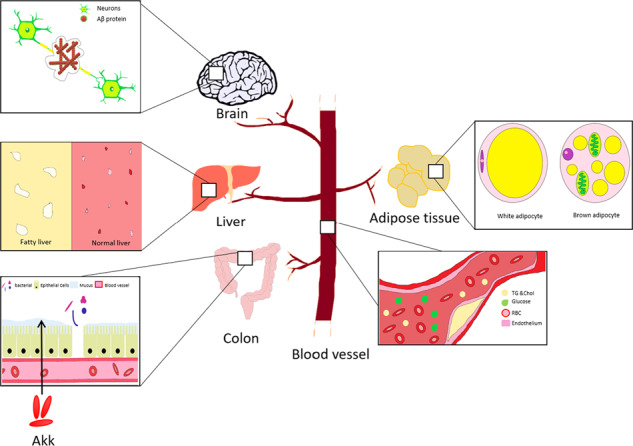

## Introduction

Alzheimer’s disease (AD) is a disease with generalized dementia, such as memory impairment, loss of visual skills, performance impairment and behavioral changes. AD is characterized by serious cognitive impairment and significant pathological changes in the brain. Amyloid β-protein (Aβ) is a peptide produced by Aβ precursor protein (APP) through proteolysis of β- and γ-secretase. Aβ40 and Aβ42 have strong toxicity, and they are easier to aggregate and cause neurotoxicity effects^1^, which is widely accepted as a key biomarker for AD. Current treatments for AD relieve only the symptoms, but cannot alleviate disease progression^2^. As such, early diagnosis is the most effective treatment method^3^. In 2010, Alzheimer’s Disease International (ADI) reported that costs of treatment and care for dementia worldwide had reached more than 818 billion dollars. Experts speculate that the population of AD patients could reach 30 million by 2050^4^, 71% of which would be concentrated in developing countries, including China, India, South Asia and the western Pacific regions.

Recently, a large number of reports have indicated that metabolic syndromes, including obesity and insulin resistance, are key high risk factors for cognitive impairment and dementia^5^. The data suggested that young people who suffered from obesity or diabetes had a greater risk of dementia^6–9^. Type 2 diabetes also greatly increased the risk of AD, and Aβ protein deposition competes with insulin for insulin receptors, causing insulin resistance^10–13^. In addition, animal experiments showed that metabolic syndrome, chronic low-grade inflammation in particular, could increase specific pathologic changes in AD^13–15^, and experts think that AD is a metabolic disease^16–18^. The pathogenesis of AD has not yet been clearly defined. Many studies have shown that the development of insulin resistance in the brain appears in AD patients, which may be caused by Aβ protein deposition, as it is associated with multiple pathological features^19,20^. Therefore, many scholars believed that a dysfunctional cerebral insulin pathway might be a partial cause of AD^21^.

*Akkermansia muciniphila* (Akk) is the only representative *Verrucomicrobia* of the human intestine microbes that can be cultured, and it can be detected easily by metagenomic analysis of the human intestine. Given that its abundance is closely related to human health, *A. muciniphila* has received increasing attention^22^. There is evidence that the abundance of *A. muciniphila* was significantly decreased in the intestines of patients with type 2 diabetes^23^ and AD model mice^24^. It has also been shown that *A. muciniphila* cannot only prevent weight gain in high-fat diet mice, but can also repair the damaged epithelial barrier integrity and improve endotoxemia^25,26^. Therefore, the specific aim of this study was to evaluate whether Akk treatment could improve the health of animal models with AD, in order to provide a new strategy for prevention and treatment of AD.

## Methods

### Animal model

APPswe/PS1dE9 (APP/PS1) double-transgenic mice and the parasite-free wild-type mice were purchased from the Model Animal Research Center of Nanjing University^27^. In order to clarify whether Akk works only under the conditions of high-fat diet, 3-month-old male APP/PS1 mice were fed ad libitum either a normal chow diet or a high-fat diet (Catalog no. D12492, Research Diet, GDMLAC) for 6 months. Moreover, in order to verify that Akk has no harmful effect in normal mice, the WT mice were divided into two groups to receive Akk administration. Taking into account, the statistical analysis and the sample size required for subsequent experiments in this study, APP/PS1 mices were randomly assigned into 4 groups (*n* = 10 per group) and WT mices were randomly assigned into two groups (*n* = 6 per group). Mice were overnight fasted and anesthetized with pentobarbital sodium. Tissues (scapular fat, cerebral cortex, hippocampus, liver, colon) were isolated and preserved after blood collection. All animal experiments were approved by the Committee on the Use of Live Animals in Teaching and Research at the University of the Southern Medical University. This study selects the sample size based on the experimental quantity requirement and the animal experiment 3R principle. All animals are grouped according to the principle of randomization and all animal experiments were performed with blinding.

### Culture and administration of *A*. muciniphila

The Chinese strain *A. muciniphila* GP01 was isolated from human stool, as previously described^28^, and cultured anaerobically in a basal mucin-based medium. The concentration of bacteria was calculated by measuring the OD values at 600 nm. The experimental APP/PS1 mice were gavaged daily for 6 months with either 5 × 10^9^ cfu of *A. muciniphila* in 200 µL sterile PBS or 200 µL sterile PBS.

### MRI

Mice were anesthetized with 1–3% isoflurane in 100% O_2_. Anesthetized mice were scanned in a 7T small animal MRI (PharmaScan70/16 US; BRUKER Biospin MRI GmbH) fitted with sensitive surface coils and amplifier. Animal welfare was provided by employing a hot water circulation system and physiological monitoring. The imaging protocol included a T2 sequence with the following parameters: TR/TE:2500/35 ms; FoV:15 × 30 mm; image size: 256 × 256 mm; Slice thickness: 0.7 × 3.4 mm; scan time: 2 min 4 s. The size of the cerebral hemispheres and lateral ventricles on both sides of the mouse can be evaluated by MRI.

### Immunohistochemistry and histology

Immunohistochemistry (IHC) was used to detect Aβ protein deposition. The paraffin sections were deparaffinized (4 μm) and dehydrated with an ethanol gradient, and antigen retrieval was accomplished by boiling in citric acid buffer for 15 min. After washing with 1 × PBS (pH 7.4), sections were incubated with 3% H_2_O_2_ to quench endogenous peroxidase activity. Sections were rinsed, incubated with 3% normal goat serum in TBS for 30 min, and incubated overnight at 4 °C with the primary antibody (β-Amyloid D12B2, Abcam, Cambridge, MA, USA). Horseradish peroxidase-conjugated secondary antibody was used to bind the primary antibody and the complex was visualized using a stable diaminobenzidine (DABI) solution. The stained sections were dehydrated with graded alcohols and cleared in xylene. Periodic acid–Schiff (PAS) staining was used to detect colonic goblet cells. The colon samples were dehydrated with an ethanol gradient (75, 85, 95, 100%) and embedded in paraffin. The sections were cut (4 μm) and processed with periodic acid (1%) for 5 min, washed with distilled water, treated with Schiff’s reagent for 5 min, and washed again. The sections were stained with Harris haematoxylin for 2 min. Hematoxylin–eosin (H&E) staining was utilized to observe hepatic steatosis. The samples were fixed in 4% paraformaldehyde and embedded in paraffin. The sections were stained with H&E as routinely described. For quantification, five sections with the same reference position were chosen from each mouse for analysis using IMAGE-PRO PLUS 6.0 imaging software.

### Biochemical assays and ELISA

Serum levels of total cholesterol (Chol), triglycerides (TG), alanine aminotransferase (ALT) and aspartate transaminase (AST) were determined using a Roche Modular P 800 Analyzer. Serum levels of diamine oxidase (DAO) (CUSABIO) and levels of Aβ40 and Aβ42 (Invitrogen) in the cerebral cortex were measured with ELISA kits.

### Glucose tolerance test

APP/PS1 mice were fasted for 12 h, and fasting blood glucose was measured. Glucose (2 g/kg) was then delivered orally and blood glucose levels were determined with a glucometer (Abbott Laboratories, Abbott Park, IL, USA) at 15, 30, 60, 90 and 120 min.

### Open-field and Y-maze tests

The open-field test arena was constructed of a plastic chamber (30 × 50 × 25 cm). The camera, connected to a computer, was placed above the chamber to monitor the distance traveled and the time devoted to the center and peripheral areas of the arena. The mice were placed in the center of the arena and allowed to explore for 10 min. Before each test, the box was cleaned with 75% alcohol. The Y-maze was done on a black-plastic board and each arm was 15 cm long, 14 cm high and 5 cm wide, and positioned at equal angles. Mice were located at the end of one arm and allowed to move freely through the maze during a 2-min session. Arm entry was considered complete when the hind paws of the mouse were completely placed in the arm. Each mouse trained 20 times a day, training for up to 3 days, until the mouse completed the test nine of ten times correctly. After 24 h, each mouse was tested ten times. The whole process was recorded, which included the total time and the correct and incorrect times. Achieving the standard: 9 or more correct responses (correct response rate ≥90%) for 10 consecutive times are set as the learning standards. Observation indicators: 1. The total time required for training, which reflects the learning ability of mice; 2. Memory retention (reproduction) test: After 24 h of learning test, test the same method 10 times. The accuracy reflects the memory ability of mice.

### Western blot analyses

Mouse hippocampi were homogenized in chilled TBS supplemented with a protease- and phosphatase inhibitor cocktail (Thermo Fisher Scientific, Waltham, MA, USA). Protein concentrations of the homogenates were determined by BCA assay (CWBIO). Samples were electrophoretically separated on 10 or 12% SDS–PAGE gels and transferred to PVDF membranes (Millipore, Bedford, MA, USA). Membranes were blocked with 5% nonfat milk in TBS containing 0.1% Tween-20 (TBS-T) for 1 h and probed with primary antibodies (Anti-UCP-1 antibody ab10983) at 4 °C overnight. The membranes were subsequently developed with the corresponding horseradish peroxidase-conjugated secondary antibody and ECL kit (Thermo Fisher Scientific). Densitometry quantification of the protein bands was analyzed using IMAGE-PRO PLUS 6.0 imaging software.

The mixture of proteins was extracted from scapular brown adipose tissues in RIPA buffer (0.5% NP40, 0.1% sodium doxycycline, 150 mM NaCl, 50 mM Tris.HCl [pH 7.4]) containing a complete protease inhibitor cocktail (Abcam). PVDF membranes were probed simultaneously with primary antibodies of UCP1 (Abcam) and β-tubulin (Abcam). The protein bands were visualized with ECL chemiluminescence reagents (Thermo Fisher Scientific) and quantified using IMAGE-PRO PLUS 6.0 imaging software.

### Real-time PCR

For analysis of Akk abundance in feces, fecal bacterial DNA was extracted by Magen Hipure Stool DNA kit. After adjusting the DNA concentration, real-time PCR was conducted using the PrimeScript™ RT reagent Kit (Cat No. RR820A, Takara, Dalian) following the manufacturer’s protocol in a LightCycle480 real-time PCR System (Roche, Germany). The procedure included pre-denaturation of DNA samples at 95 °C for 30 s, then 40 cycles of denature at 95 °C for 5 s and anneal at 60 °C for 30 s, melted at 95 °C for 5 s and 60 °C for 1 min, finally cooled at 50 °C for 30 s. The fluorescent product was detected in the last step of each cycle. Relative quantification was calculated with 2^−ΔΔCt^, the results were normalized to 16 S rRNA gene.

The primers were: *Akkermansia muciniphila* gene,

Forward 5′-AGCCTGAACGCATTATCCGC-3′;

Reverse 5′-AGGGCAGTTCTTCCAGCGTA-3′.

16S rRNA gene,

Forward 5′-GTGYCAGCMGCCGCGGTAA-3′;

Reverse 5′-GGACTACNVGGGTWTCTAAT-3′.

### Statistical analysis

All samples were involved in statistical analysis. All analyses were performed with the Statistical Package for Social Sciences version 19.0 (SPSS) (IBM Corp, Armonk, NY, USA). Data were expressed as mean ± SEM. Statistical significance was determined by Student’s *t*-test (for comparison of two experimental groups) or one-way ANOVA (for comparison of three or more experimental groups). Comparisons with *p* < 0.05 were considered statistically significant.

## Results

### Akk effectively regulated the glucose homeostasis and damage to the intestinal barrier in APP/PS1 mice

**Fig. 1:**
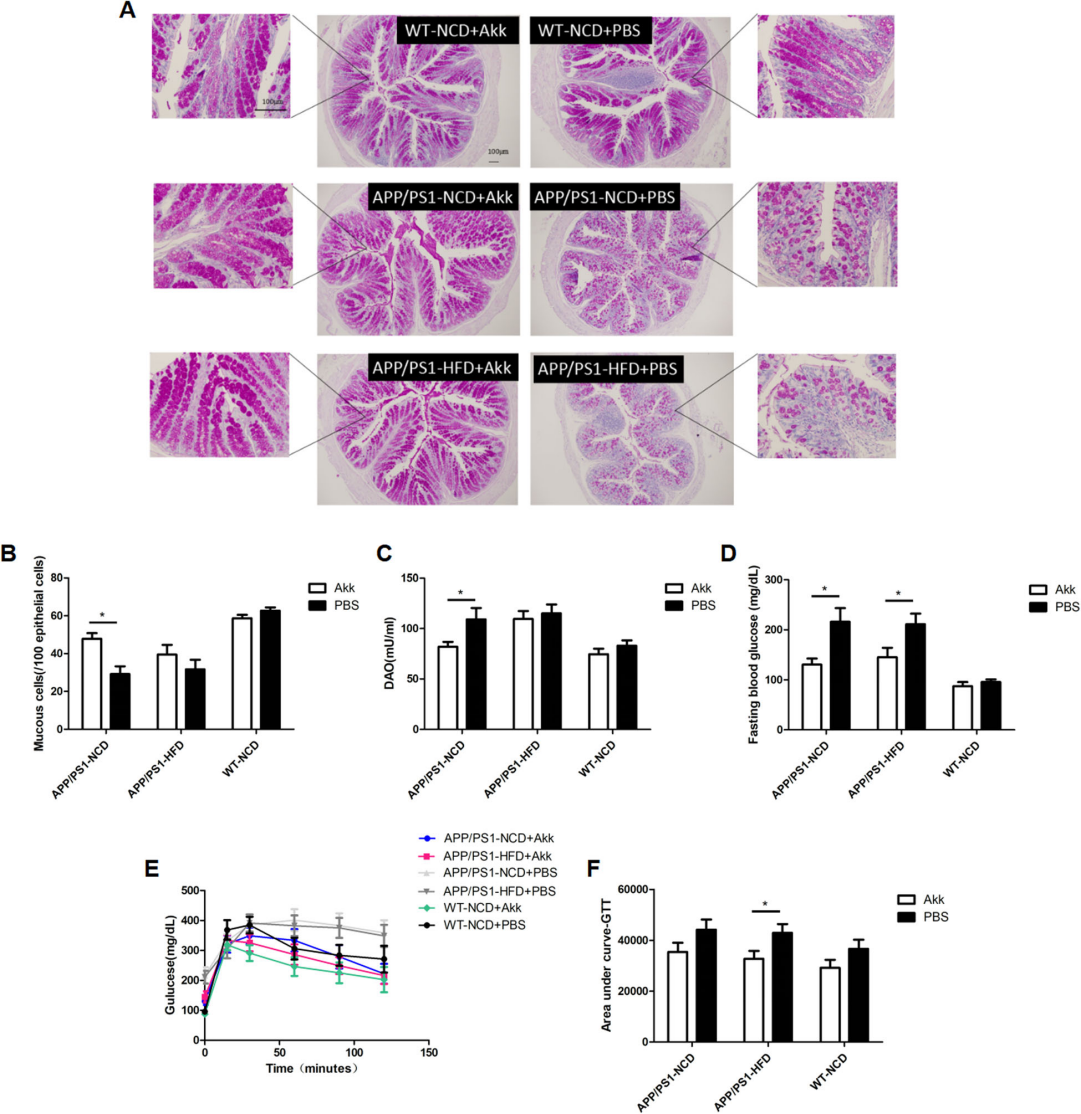
*Akkermansia muciniphila* ameliorated glucose homeostasis and restored intestinal barrier function. **a**, **b** Quantification of colonic mucus cells identified by periodic acid–Schiff reaction. **c** Serum DAO levels of the in vitro gut permeability assay. **d** Fasting serum glucose profile in the mice. **e**, **f** The mean area under the curve (AUC) was measured during an oral glucose tolerance test (OGTT). Data are presented as the mean ± SEM, *N* = 6–10 per group. *p < 0.05; **p < 0.01; ***p < 0.001.

To assess the effects of Akk on the intestinal barrier and glucose homeostasis in an AD model mouse, we compared in the Akk-treated and PBS-treated APP/ PS1 mice fed either a normal chow diet (NCD) or a high-fat diet (HFD). In APP/PS1 mice treated with PBS, we found that the fasting blood glucose level was higher, and OGTT results also showed an increase in area of the AUC (Fig. 1d–f, *p* < 0.05, respectively). In striking contrast, significantly reduced levels of fasting blood glucose in APP/PS1 mice appeared after Akk treatment, and impaired glucose tolerance was improved, but only the high-fat diet group showed statistical differences (Fig. 1d–f, *p* < 0.05, respectively). These results demonstrated that in APP/PS1 mice, Akk reduced fasting blood glucose levels and regulated glucose metabolism disorders.

Next, we explored the possible role of Akk in intestinal barrier function in APP/PS1 mice. Diamine oxidase (DAO) is a highly active intracellular enzyme in the intestinal upper villus in human and mammals, has a role in the metabolism of histamine and various polyamines, and is closely related to nucleic acid and protein synthesis in mucosal cells (Fig. 1a, b, *p* < 0.05). DAO can reflect the integrity of the intestinal mechanical barrier and the degree of damage. Our results showed that the colonic mucus cells in PBS-treated APP/PS1 mouse were greatly reduced, and serum DAO levels were significantly increased, as demonstrated by PAS staining and ELISA (Fig. 1c, *p* < 0.05). The results demonstrated that intestinal barrier function in APP/PS1 mice was disorder. However, after treatment with Akk, there was no significant decrease in colonic mucus cells, and serum DAO levels were significantly lower than those in the PBS group. Only the NCD groups showed statistical difference, indicating that Akk could repair the intestinal barrier dysfunction in APP/PS1 mice.

### Akk effectively relieved hyperlipidemia and whitening of brown adipose tissue in APP/PS1 mice

**Fig. 2:**
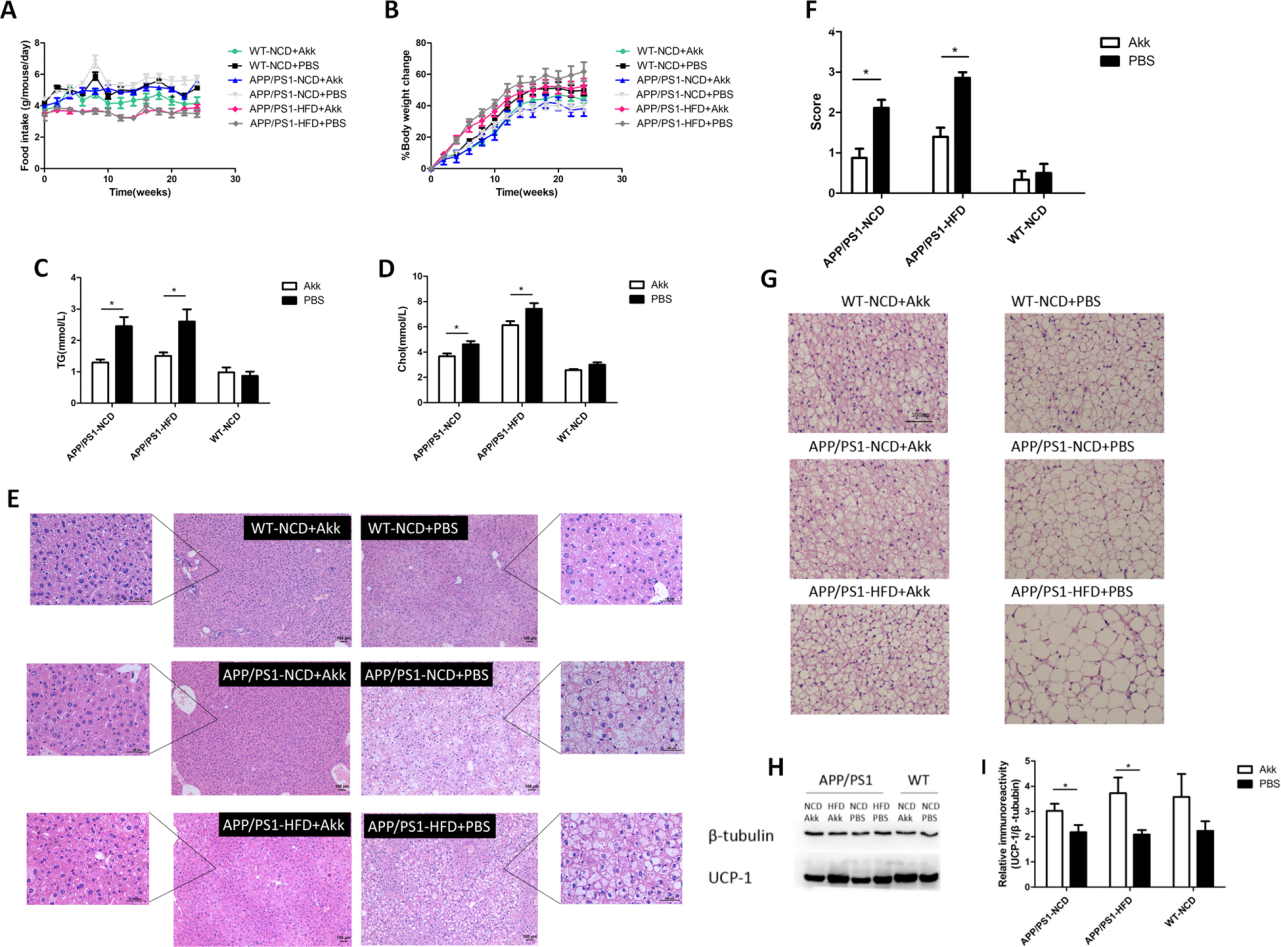
*Akkermansia muciniphila* ameliorated lipid metabolism disorders. **a** Body weight gain and **b** daily food intake per mouse. **c** Serum TG and **d** Chol levels were measured in the mice. **e**, **f** Histological assessment of hepatic steatosis with representative pictures of H&E stained liver sections. **g** Representative H&E images of scapular adipose tissue deposits. **h** Representative western blot of scapular BAT lysates in the six groups. **i** UCP1 expression was quantified using Image-Pro Plus 6.0 software and UCP1 content of brown adipose tissue was analyzed by immunohistochemistry. Data are presented as the mean ± SEM, *N* = 6–10 per group. *p < 0.05; **p < 0.01; ***p < 0.001.

We explored the effects of Akk on lipid metabolism in AD model mice. In APP/PS1 mice, there was no obvious change in food intake or body weight gain between the Akk and PBS treatment groups (Fig. 2a, b). In addition, 9-month-old APP/PS1 mice developed significant lipid metabolism disorders, evidenced by increasing levels of serum Chol and TG (Fig. 2c, d, *p* < 0.05, respectively), and fat accumulation in the liver, which was similar between the high-fat diet group and normal diet group. Interestingly, lipid metabolism disorders in APP/PS1 mice treated with Akk were significantly improved, showing a decrease in serum Chol and TG levels, and hepatic fat scores showed ameliorative hepatic steatosis (Fig. 2e, f, *p* < 0.05, respectively). It is worth noting that there were no differences in serum ALT and AST levels between six groups (Supplementary Fig. 2). Brown adipose tissue (BAT) possessed the anti-obesity potential, which was characterized by large numbers of mitochondria, where uncoupling protein 1 (UCP1) possesses the ability to utilize free fatty acids and glucose for heat generation (non-shivering thermogenesis). Because the cells are rich in tiny lipid droplets, brown adipocytes take on a multilocular appearance after H&E staining. We found that the scapular brown adipocytes in APP/PS1 mice changed from multilocular adipocytes to unilocular adipocytes, shown as whitening of the brown fat. After Akk treatment, the whitening phenomenon of brown fat was obviously weakened, and the number of unilocular adipocytes in scapular brown fat tissue was also reduced (Fig. 2g). Moreover, after Akk treatment, the expression of UCP1 in the brown fat of APP/PS1 mice was increased, suggesting a more active themogenesis. High-fat diet aggravated the whitening of brown adipocytes in APP/PS1 mice, but did not reduce the thermogenic activity in brown fat (Fig. 2h, j, *p* < 0.05, respectively). These results indicated that Akk improved hyperlipidemia and hepatic steatosis in APP/PS1 mice, alleviated the whitening of brown fat and maintained thermogenic activity. In summary, Akk supplementation can regulate lipid metabolism disorders in APP/PS1 mice.

### Akk reduced Aβ plaque deposits and Aβ levels in brains of APP/PS1 mice

**Fig. 3:**
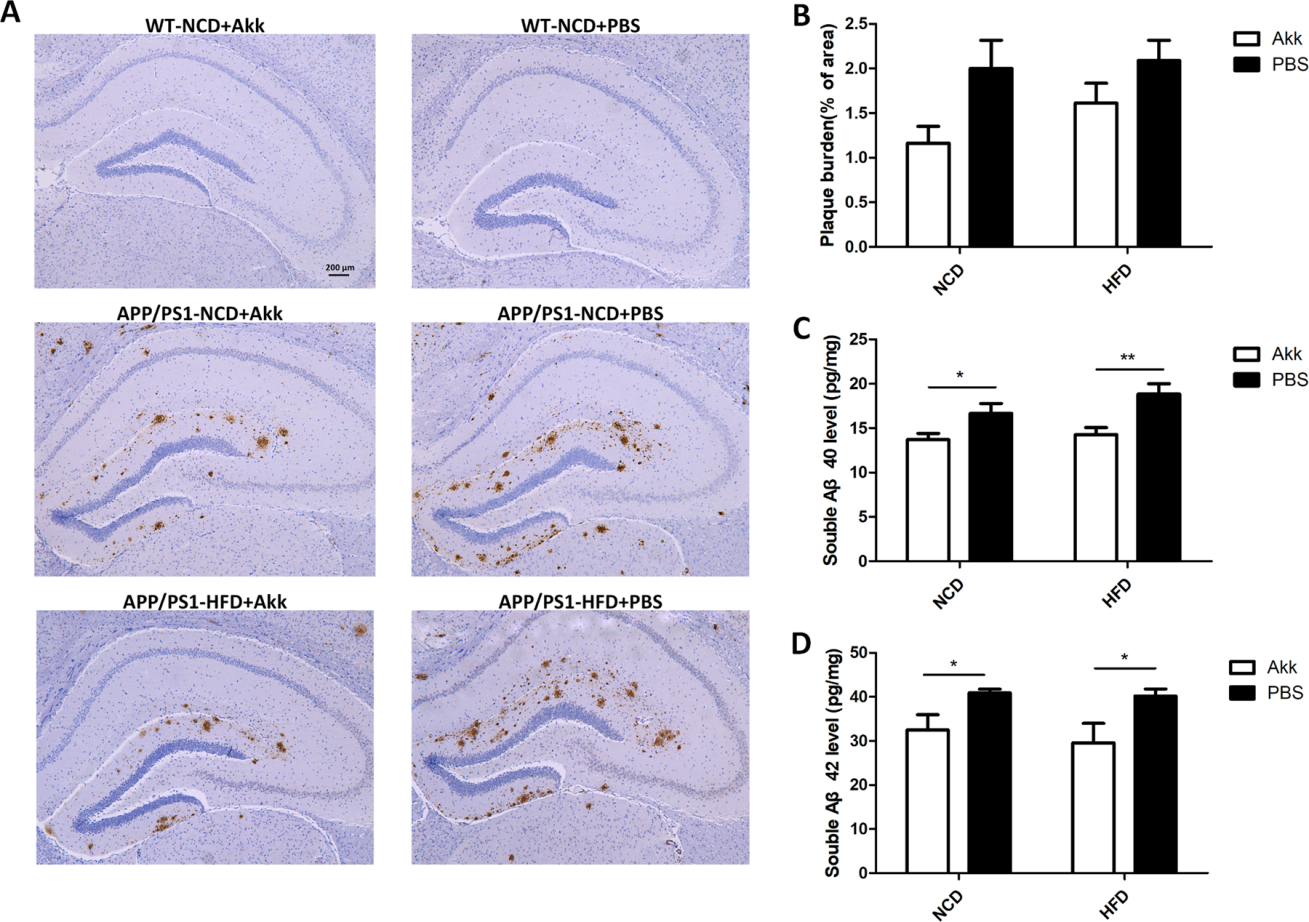
Akkermansia *muciniphila* reduced Aβplaque deposits and Aβ levels in brains of APP/PS1 mice. **a** Representative IHC images of the hippocampus from the mice. **b** Statistical analyses of the plaque burden. **c** Soluble Aβ40 and **d** Aβ42 were analyzed by ELISA in the cortex of APP/PS1 mice. Data are presented as the mean ± SEM, *N* = 6–10 per group. *p < 0.05; **p < 0.01; ***p < 0.001.

Analysis of Aβ protein deposition by immunohistochemistry showed that APP/PS1 mice had more Aβ plaques in the hippocampus (Fig. 3a).The area of Aβ protein deposition increased in APP/PS1-HFD-PBS mice, but was reduced when treated with Akk, although there was no statistically significant difference (Fig. 3b) In addition, we also analyzed soluble Aβ 40 and Aβ 42 proteins via ELISA. In APP/PS1-Akk mice, we detected a significant reduction in soluble Aβ 40 and Aβ 42 levels compared with APP/PS1-PBS mice (Fig. 3c, d, *p* < 0.01; *p* < 0.05, respectively). MRI showed that there was not significant atrophy in the mouse brains (Supplementary Fig. 3), indicating that differences between the APP/PS1-Akk mice and the APP/PS1-PBS mice were not caused by changes in brain structure. The results suggested that Akk intervention could reduce Aβ plaque deposits and Aβ levels in the brains of APP/PS1 mice independent of changes in brain structure.

### Akk improved impaired cognition and anxiety-related behaviors in APP/PS1 mice

**Fig. 4:**
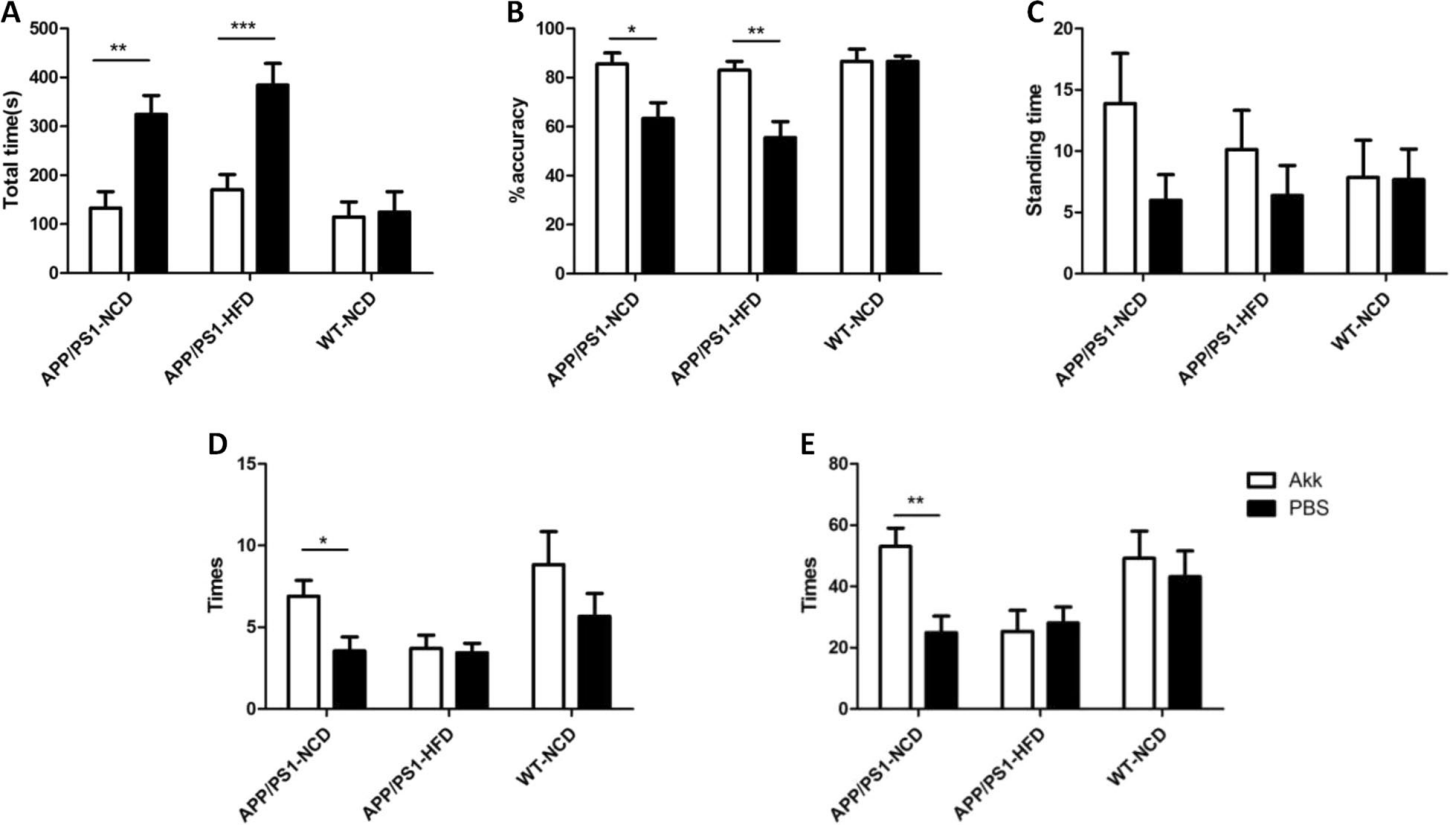
*Akkermansia muciniphila* ameliorated cognitive impairment and anxiety-related behaviors in APP/PS1 model mice. **a**, **b** The total time of learning and accuracy rate after 24 h of training in the Y-maze tests. **c**–**e** In the open-field tests, the time spent in the center and the total times of clearing and standing on the hind legs were monitored for each mouse. Data are presented as the mean ± SEM, *N* = 6–10 per group. *p < 0.05; **p < 0.01; ***p < 0.001.

In order to investigate the learning and memory capabilities of APP/PS1 mice, the Y-maze test was performed. Before the intervention, all mice received the Y-maze test for a maximum of 3 days. However, the APP/PS1-PBS mice spent more time than WT-PBS mice when training. After 6 months of Akk treatment, APP/PS1 mice required significantly shorter learning time than PBS-treated group (Fig. 4a, *p* < 0.001). These data indicated that Akk could alleviate the damage to learning ability in APP/PS1 mice. After 24 h of practice in the Y-maze, each mouse was controlled for 10 times. The APP/PS1-PBS mice had rates of correct maze solving that were significantly lower than those in the other groups (Fig. 4b, *p* < 0.01), which indicated that Akk was effective in improving the impairment of memory capacity in APP/PS1 mice.

In the open-field test, there were no significant differences when comparing the proportion of time spent in the central district among all groups (Fig. 4c). The clearing times of the APP/PS1-PBS mice were lower than those of the WT-PBS mice (Fig. 4d, *p* < 0.05), but the clearing times were significantly increased in APP/PS1 mice treated with Akk than in the PBS treatment group. Only mice fed a normal diet showed a significant difference, which indicated that the gavage with Akk may have played a role in improving the ability of the mice to remain attentive. In addition, the duration of standing on the hind legs in the WT mice was higher than in the APP/PS1 mice when compared to the PBS intervention group, which did not show a significant difference after Akk treatment (Fig. 4e, *p* < 0.01). In the APP/PS1 mice, Akk intervention significantly reduced the duration of standing in mice fed a normal diet, suggesting that Akk treatment could also help maintain curiosity regarding the surrounding environment, although the effect did not appear in the mice fed a high-fat diet. These data indicated that Akk could reduce the damage to spatial learning ability in APP/PS1 model mice.

## Discussion

Recently, increasing numbers of studies have reported that a close relationship exists between gut microbiota and neurological disorders, which has been called the gut-brain axis^29^. Studies have shown that intestinal microbes have important implications in AD^30^. AD is a progressive degenerative disease of the central nervous system, mainly characterized as cognitive impairment, memory dysfunction, decreased self-care ability and decreased behavioral ability. There were major differences in the intestinal microflora composition between AD patients and normal subjects, suggesting that microbial composition may have a role in the development of brain Aβ amyloidosis^24^. The reduction in the abundance of *Akkermansia* was reported to be associated with obesity, type 2 diabetes, dysfunction of the intestinal barrier and other metabolic syndromes^31–33^, most of which are important risk factors for AD. It has been further reported that the abundance of Akk in APP/PS1 mice decreased with age^24^. These reports suggest that supplementation with Akk could be a new strategy for the prevention and treatment of AD. In the present study, with age, the Akk abundance decreased in APP/PS1 mice (Supplementary Fig. 1), and APP/PS1 mice showed a significant increase in fasting blood glucose levels and insulin resistance at 9 months of age, the mucus cells in the colon were significantly decreased and the serum DAO level was increased, indicating damaged intestinal barrier function. In addition, obvious impairment of cognitive function and learning memory ability, and an apparent accumulation of Aβ protein in the brain were observed in 9-month-old APP/PS1 mice.

In the past decades, researchers believed that metabolic syndromes, including insulin resistance and obesity, were important risk factors for cognitive impairment and dementia^5^. Studies had shown that insulin receptor expression was defective in the brains of AD patients and AD model animals. Studies also showed that IR substrate-1 (IRS1) and IR substrate-2 (IRS2) expression were decreased, and the levels of inactivated serine-phosphorylated IRS1 were increased, resulting in insulin resistance in the brain. Moreover, intestinal barrier destruction and endotoxemia were observed in AD model animals^20^. Bacterial-derived LPS and amyloid protein can aggravate intestinal permeability, resulting in increased expression of cytokines and pro-inflammatory factors such as interleukin 17A (IL-17A) and IL-22, which can cross the intestinal barrier and blood brain barrier and cause an immune response in brain^34^. These studies indicated that AD may be a metabolic disease, and some scholars refer to it as type III diabetes^5^. Our study found that Akk could improve impaired fasting glucose and insulin resistance in AD model mice, increase the number of colonic mucus cells, decrease serum DAO levels and repair damaged intestinal barriers. Moreover, we found that compared to mice in the normal chow diet group, AD mice in the high-fat diet group have shorter colons, heavier weight, and higher cholesterol levels, liver steatosis is more severe (Supplementary Fig. 4). On the basis of our experimental results, Akk has a significant effect on alleviating the disease of AD mice, no matter in the high-fat diet group or the normal chow diet group, indicating that Akk has a protective role in AD disease under different conditions of abnormal lipid metabolism.

Epidemiological and clinical studies have shown that adults with obesity or diabetes have a much higher risk of developing dementia in the future. Obesity, insulin resistance and hyperlipidemia are forms of lipid toxicity, leading to inflammation, which may have a key role in neurological dysfunction. Cognitive impairment in the CNS could be attributed to changes in hippocampus structure and function in a portion of obesity-induced patients^35^. Similarly, brain damage might also promote the development of obesity. We found that APP/PS1 mice showed significantly increased food intake, and the emergence of lipid metabolism disorders, such as increased levels of total cholesterol and triglycerides, liver steatosis, scapular brown adipocytes whitening, which were aggravated when treated with HFD. It is important to note that the above symptoms in APP/PS1 mice were improved after the mice were treated with Akk. Scapular brown adipocyte whitening could be seen in obese and ageing mice^36–39^, which was also observed in APP/PS1 mice in the course of our experiments. Our results demonstrated that Akk alleviated whitening of scapular brown adipocytes caused by a high-fat diet and aging. The reason may be that Akk can promote the utilization of free fatty acids and glucose in circulating blood, which can serve as a substrate to maintain the thermogenic activity of brown adipose tissue, so as to ensure the morphology and function of brown adipocytes^37^. Mice treated with PBS were unable to make full use of free fatty acids and glucose, resulting in severe accumulation of fat and elevated levels of blood glucose, brown adipocytes gradually replaced by white adipocytes, decreased thermogenesis and reduced energy consumption. These mice became obese and displayed liver steatosis. This hypothesis may also explain, to some extent, how Akk improved liver steatosis and metabolic syndrome. Consistent with this hypothesis, we found that Akk increased the expression of UCP1, which is a protein specifically expressed in brown adipose, which reflects the cellular thermogenic activity. The levels of UCP1were higher in mice treated with HFD. This phenomenon might be explained by diet-induced thermogenesis (DIT)^40,41^.We proffer that HFD stimulated thermogenesis in brown adipose tissue (BAT), and the mice displayed elevated BAT activity. Further study is needed to explore and verify this mechanism, however.

The main pathological features of AD are the formation of senile plaques (SP) and neurofibril tangles (NTFs) in the brain^42^. Moreover, the main component of SP is β-amyloid (Aβ), which has a crucial role in the occurrence and development of AD, and is a common pathway for various factors to induce AD^43^. Aβ protein has an important role in the pathogenesis of AD. Self-clearing and degradation barriers of Aβ protein lead to protein plaques. The Aβ protein can reduce the clearance of Aβ protein by macrophages, thereby producing an immune response^44^. The Aβ protein can also promote neuronal apoptosis. There was a major difference in the composition of intestinal microflora between AD patients and normal subjects, suggesting that microbial composition may have a role in the development of brain Aβ amyloidosis^24^. It is worth mentioning that, in our study, Aβ protein deposition was reduced in the hippocampus and the cerebral cortex of APP/PS1 mice treated with Akk.

APP/PS1 mice also showed reduced attention to themselves and the environment after evaluation via the open-field test in our study, in agreement with previous studies^27^, and these phenomena were alleviated by Akk treatment. The results of the Y-maze test showed that a high-fat diet further aggravated the impairment of learning and spatial memory ability in APP/PS1 mice. However, these symptoms were significantly improved with Akk treatment. These results suggest that Akk may increase mucus cells and promote the secretion of mucus to repair the impaired intestinal barrier, thereby reducing levels of LPS and other pro-inflammatory substances, improving metabolic levels, relieving insulin resistance in the brain, reducing the deposition of Aβ protein, and protecting the nerves from damage.

In general, Akk treatment effectively improved intestinal barrier dysfunction, glucose and lipid metabolic disorders, and relieved cognitive impairment by reducing the level of Aβ plaque deposition in the brain. These findings suggest that the ameliorative effect of Akk treatment on cognitive function can be attributed to the repair of metabolic disorders and, concomitantly, reduced incidence of Aβ pathology. Our findings will provide a new insight that Akk supplementation is expected to be a new strategy for the prevention and treatment of AD.

## Supplementary information

Supporting Information

Supplementary Figure 1

Supplementary Figure 2

Supplementary Figure 3

Supplementary Figure 4
