# Supplementary material for: Protective effects of Akkermansia muciniphila on cognitive deficits and amyloid pathology in a mouse model of Alzheimer’s disease
Source: Nutr Diabetes. 2020 Apr 22;10:12. doi: 10.1038/s41387-020-0115-8 (PMC7176648; doi:10.1038/s41387-020-0115-8)
Supplement: Supplementary file 1 — Supporting Information [file 41387_2020_115_MOESM1_ESM.pdf]

Supporting Information

Protective Effects of *Akkermansia muciniphila* on Cognitive Deficits and Amyloid Pathology in a Mouse Model of Alzheimer's Disease

Zihao Ou 1, Lulu Deng1, Zhi Lu1, Feifan Wu1,2, Wanting Liu1,Dongquan Huang1, Yongzheng Peng1, 3#

[1] Department of Laboratory Medicine, Zhu Jiang Hospital, Southern Medical University, Guangzhou, Guangdong 510282, China;

[2] Jiangmen Central Hospital, Affiliated Jiangmen Hospital of Sun Yat-sen University, Jangmen, Guangdong 529000, China;

[3] Transfusion Medicine, Zhu Jiang Hospital, Southern Medical University, Guangzhou, Guangdong 510282, China.

Author to whom correspondence should be addressed; Yongzheng Peng, Department of Laboratory Medicine, Zhu Jiang Hospital, Southern Medical University, Guangzhou, Guangdong, China, 510282, yzpeng1981@126.com, +86-020-61643489, +86-020-61643010.

Email of all author:

Zihao Ou: ouzihao2015@126.com

Lulu Deng: 869765948@qq.com

Zhi Lu: 942135298@qq.com

Feifan Wu: 424176302@qq.com

Wanting Liu: 1114202711@qq.com

Dongquan Huang:735134325@qq.com

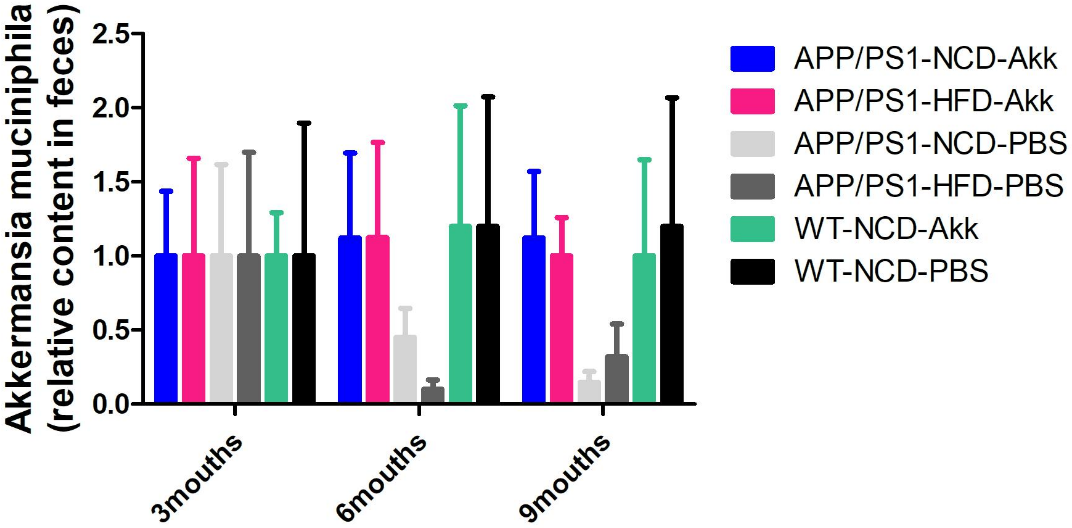

Supplementary Figure 1 *Akkermansia muciniphila* relative content in mice. Data are presented as the mean ± SEM, N=6-10 per group. *P < 0.05; **P < 0.01; ***P < 0.001.

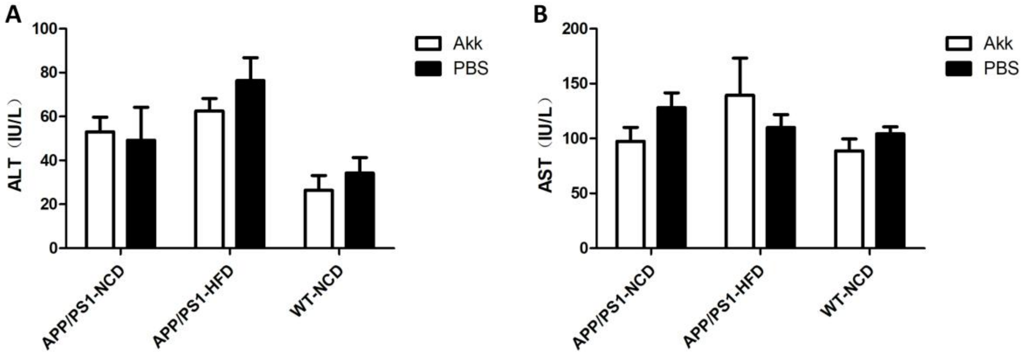

Supplementary Figure 2 Serum ALT and AST levels in the mice. Data are presented as the mean ± SEM, N=6-10 per group. *P < 0.05; **P < 0.01; ***P < 0.001.

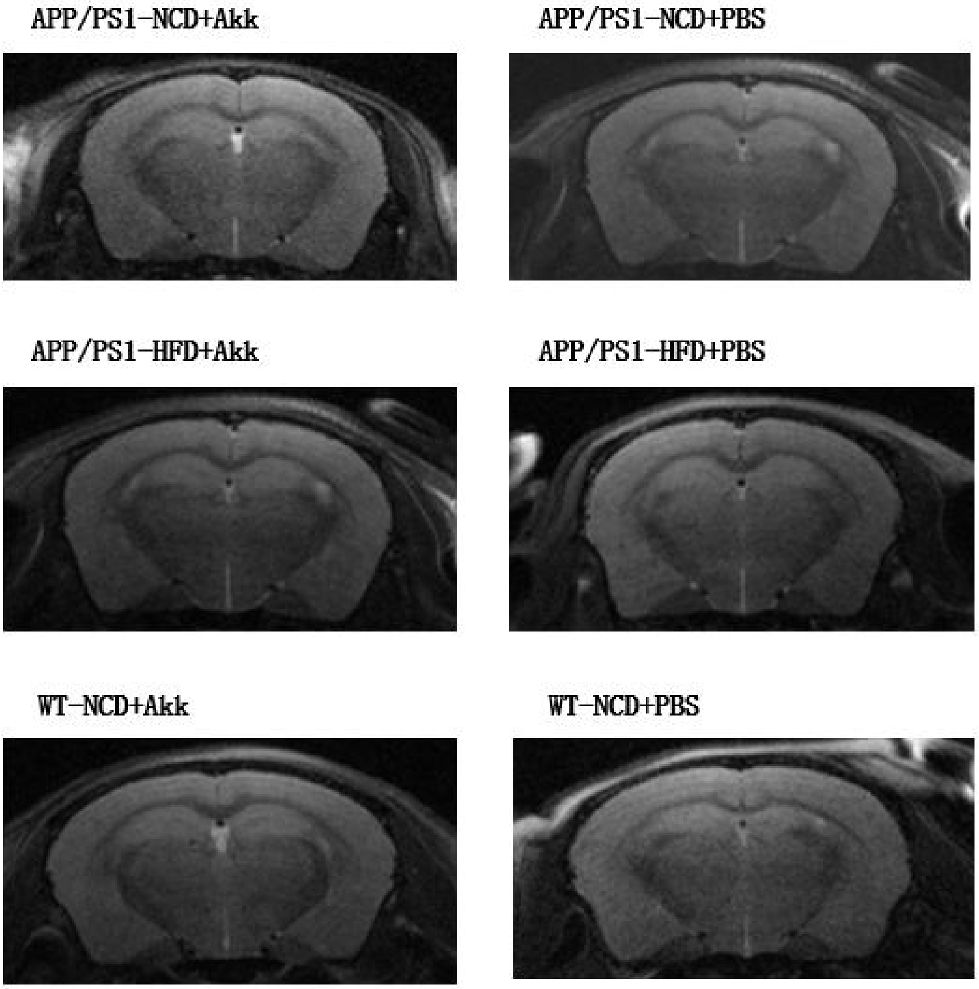

Supplementary Figure 3 Representative MRI images of mice brains. Data are presented as the mean ± SEM, N=6-10 per group. *P < 0.05; **P < 0.01; ***P < 0.001.

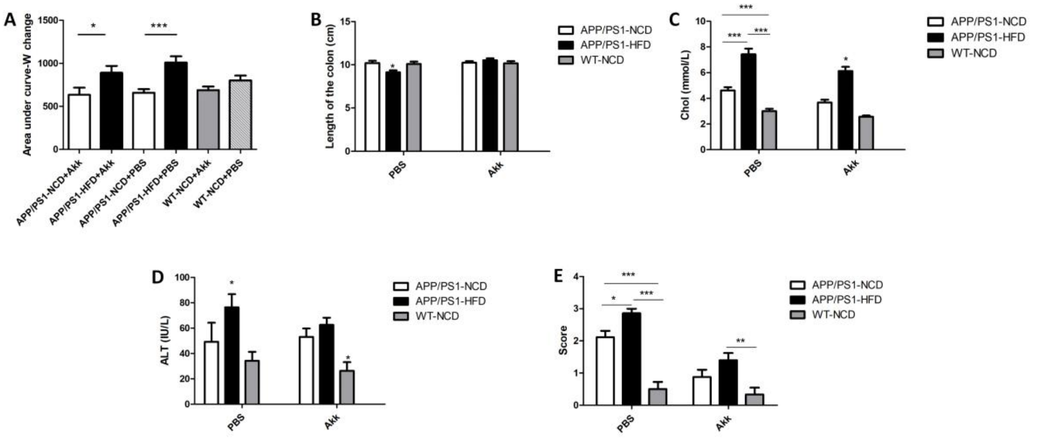

Supplementary Figure 4 (A,B) Body weight gain and length of colon were measured per mouse. (C,D) Chol and ALT levels were measured in the mice. (E) Histological assessment of hepatic steatosis with representative pictures of H&E stained liver sections.Data are presented as the mean ± SEM, N=6-10 per group. *P < 0.05; **P < 0.01; ***P < 0.001.
