# Supplementary figures and images for: Protective effects of Akkermansia muciniphila on cognitive deficits and amyloid pathology in a mouse model of Alzheimer’s disease
Source: Nutr Diabetes. 2020 Apr 22;10:12. doi: 10.1038/s41387-020-0115-8 (PMC7176648; doi:10.1038/s41387-020-0115-8)

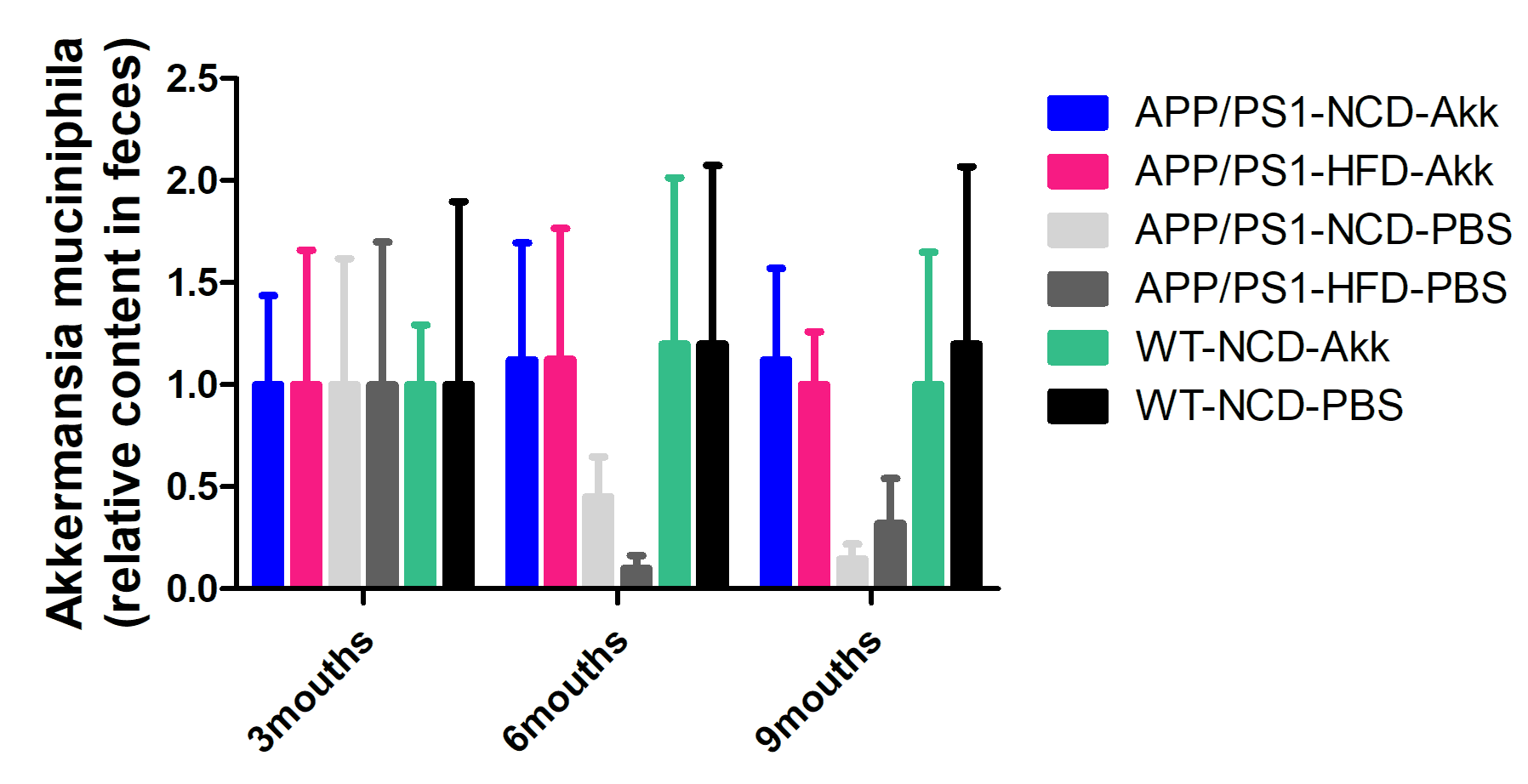

Supplement: Supplementary file 2 — Supplementary Figure 1 [file 41387_2020_115_MOESM2_ESM.tif]

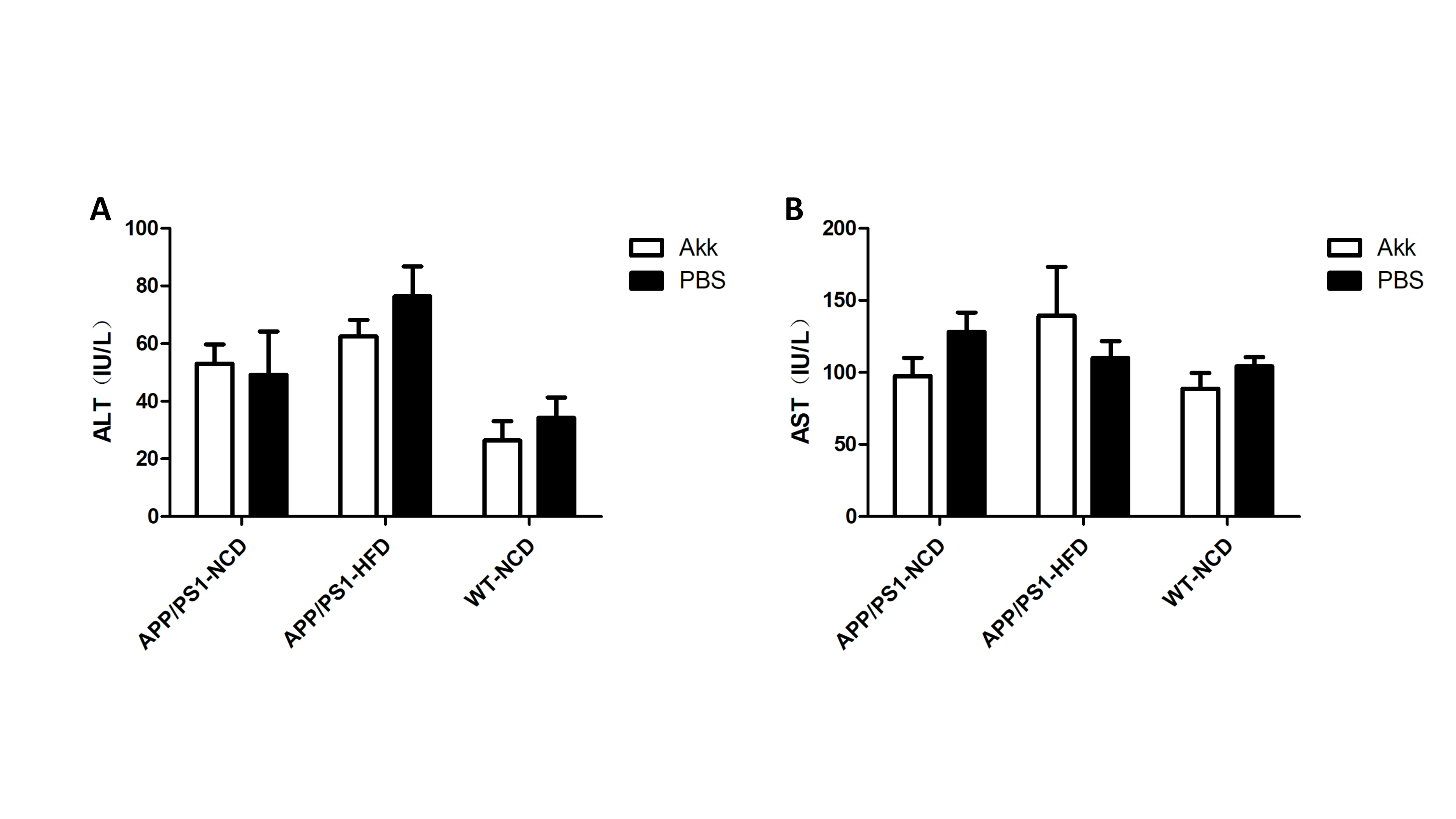

Supplement: Supplementary file 3 — Supplementary Figure 2 [file 41387_2020_115_MOESM3_ESM.tif]

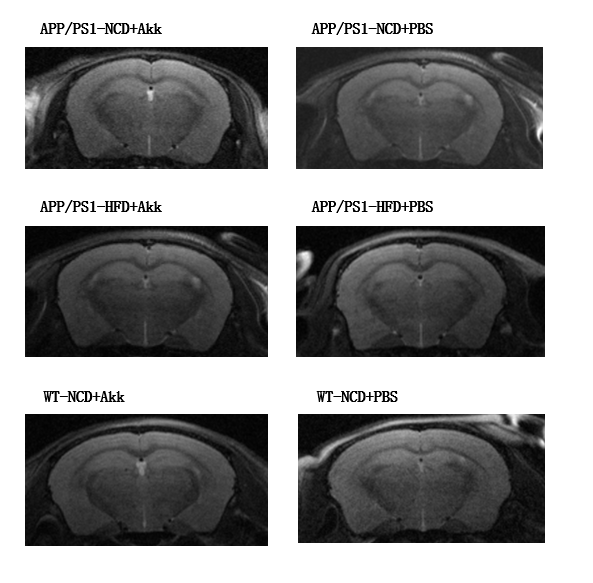

Supplement: Supplementary file 4 — Supplementary Figure 3 [file 41387_2020_115_MOESM4_ESM.tif]

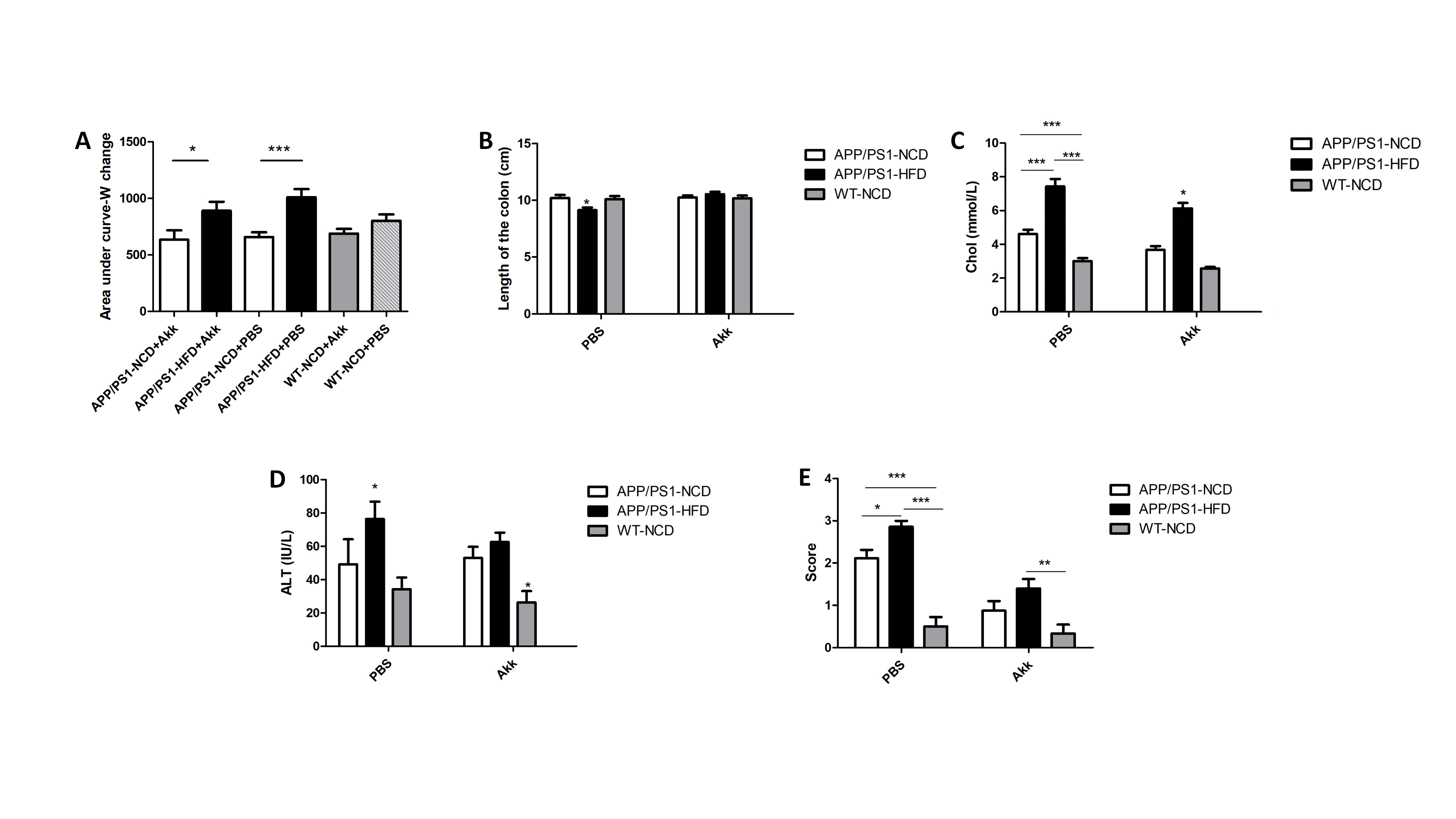

Supplement: Supplementary file 5 — Supplementary Figure 4 [file 41387_2020_115_MOESM5_ESM.tif]
